# A Mobile Phone Text Messaging Intervention to Manage Fatigue for People With Multiple Sclerosis, Spinal Cord Injury, and Stroke: Development and Usability Testing

**DOI:** 10.2196/40166

**Published:** 2022-12-21

**Authors:** Kerri A Morgan, Alex W K Wong, Kim Walker, Rachel Heeb Desai, Tina M Knepper, Pamela K Newland

**Affiliations:** 1 Program in Occupational Therapy St. Louis School of Medicine Washington University St. Louis, MO United States; 2 Center for Rehabilitation Outcomes Research Shirley Ryan AbilityLab Chicago, IL United States; 3 Department of Physical Medicine and Rehabilitation Feinberg School of Medicine Northwestern University Chicago, IL United States; 4 Department of Medical Social Sciences Feinberg School of Medicine Northwestern University Chicago, IL United States; 5 Goldfarb School of Nursing Barnes Jewish College St. Louis, MO United States

**Keywords:** fatigue, disability, mobile health, mHealth, patient activation

## Abstract

**Background:**

Fatigue significantly affects daily functioning in persons with disabilities. Fatigue management can be challenging, and the information provided during routine physician visits to manage fatigue can be overwhelming. One way to address fatigue is to increase knowledge, skills, and confidence for self-management (ie, patient activation). Self-management programs have shown promising effects in targeting fatigue in persons with disabilities. However, satisfaction with self-management programs is low for persons with disabilities, and tailoring interventions to personalized needs has been recommended. SMS text messaging is increasingly being used to implement health behavior change interventions in a person’s natural environment. Little has been done to link mobile health approaches with patient activation and self-management to address fatigue in persons with disabilities.

**Objective:**

This study aimed to develop and test a mobile phone–based fatigue self-management SMS text messaging intervention targeting patient activation in 3 groups of persons with disabilities: persons with multiple sclerosis, persons who had a stroke, and persons with a spinal cord injury.

**Methods:**

We used evidence-based resources and input from a consumer advisory board (CAB; composed of 2 participants from each of the 3 disability groups) and a neurologist to develop the intervention. The study was conducted using a 4-step process: development of the initial SMS text messaging library and categorization of the content into 9 content areas, review and modification of the SMS text messages by the neurologist and CAB, integration of the content library into a digital platform, and utility testing by CAB members.

**Results:**

A total of 6 CAB participants rated SMS text messages covering 9 domain areas of fatigue self-management with good clarity (mean ratings=3.5-5.0 out of 5) and relevance (mean ratings=3.2-5.0 out of 5). Overall, SMS text messaging content was reported by CAB participants as helpful, clear, and well suited for a mobile health intervention. The CAB reached consensus on the time of day that SMS text messages should be sent (morning) and their frequency (once per day). This feedback led the research team to narrow down the program to deliver 48 SMS text messages, 1 per day, Monday through Thursday only, a total of 4 SMS text messages per week, over a 12-week period. The final set of SMS text messages was programmed into a digital platform with a predefined delivery schedule. The usability of the intervention was high, with 55 (83%) out of 66 responses endorsing the highest rating.

**Conclusions:**

This study demonstrates a step-by-step process for developing a fatigue self-management SMS text messaging intervention for persons with disabilities. For this population, whose access to health services is often limited, this intervention provides an alternative delivery model to increase access to fatigue information and deliver content that aligns with the person’s needs.

## Introduction

### Background

Fatigue significantly impacts the daily functioning of persons with disabilities [1] and decreases participation in major life activities [2-4]. Persons with disabilities reported fatigue as a common and burdensome symptom. Approximately 50% to 80% of people with multiple sclerosis (MS), a spinal cord injury (SCI), or a stroke experience fatigue [2,5,6], a rate 2 to 3 times more prevalent than in the general population and significantly higher than in older adults with other medical conditions [7]. Particularly for persons with disabilities, fatigue is related to other symptoms such as sleep or pain [8] and negatively impacts psychological well-being and quality of life [9-11]. Fatigue also leads to absenteeism and affects the work and productivity of individuals with physical disabilities and chronic disease [12-14]. Fatigue is estimated to cost employers US $136 billion annually in health-related lost work and productivity in the general population [15].

The management of chronic symptoms can be challenging. Self-management programs have shown promising effects in improving self-efficacy and the ability to manage symptoms of persons with disabilities [1,16,17]. Although fatigue is common and distressing across a wide range of chronic disability conditions, no fatigue self-management intervention can be used across different disability groups [18]. A previous report indicated that fatigue management could benefit from a general transdiagnostic approach, with the goal of focusing on individual needs rather than a specific disease [19]. Thus, developing a general fatigue self-management intervention that can be applied across various groups may benefit a larger population living with various disabilities. Satisfaction with current self-management interventions for persons with disabilities is low because the experience of fatigue is unique to the patient experience, and tailoring toward personalized needs has thus been recommended [20]. One possible solution to address these challenges is to increase patient activation—knowledge, skills, and confidence for self-management [21]. Evidence suggests that increasing levels of patient activation improve health outcomes and care experiences and reduce health care costs [22-24]. Mobile health (mHealth) tools, especially SMS text messages using mobile phones, appear to be effective in improving activation and self-management behaviors in chronic disabling conditions [25-27]. SMS text messaging interventions are also increasingly being used to implement self-management programs for persons with disabilities because of their high reach, high accessibility, and relatively low-cost communication strategies [16,28-32]. However, there is limited research linking mHealth approaches with patient activation and self-management to address fatigue in persons with disabilities.

### Objective

This study aimed to describe the process of developing and testing the utility of a fatigue self-management SMS text messaging intervention based on patient activation for persons with disabilities. Engaging end users in designing a fatigue self-management SMS text messaging intervention in the early phase of technology development may improve user experiences, accessibility, and usability of the technology [33]. Thus, the design of this study incorporated individuals with MS, SCI, and stroke to provide valuable feedback for developing the fatigue self-management SMS text messaging intervention.

## Methods

### Participants

This study included an interdisciplinary investigator team with unique clinical expertise in MS, SCI, and stroke. All investigators had experience in disability research to develop the fatigue management SMS text messaging intervention for persons with disabilities. The investigator team organized a consumer advisory board (CAB) that included 2 persons from each disability group (MS, SCI, and stroke). Inclusion criteria for CAB participants were as follows: (1) aged >18 years, (2) had a disability for at least 1 year, (3) reported fatigue that was well managed, (4) had the ability to read and speak English at an eighth-grade level, and (5) were willing to use their own mobile phone and SMS to trial the SMS text messaging intervention. Exclusion criteria included the following: (1) evidence of an acute condition (eg, relapse), (2) sleep apnea, (3) inability to provide consent, (4) terminal cancer, and (5) pregnancy. We used purposive sampling to maximize different ages, genders, races, and ethnicities in an attempt to represent a diverse spectrum of persons with disabilities. The final sample was composed of 6 persons with disabilities, which was deemed by the investigator team as large enough for utility testing but small enough to conduct advisory board sessions in a focus group format [34,35]. A larger sample size is required for future pilot testing. This study also included a physiatrist who had provided medical rehabilitation care for >20 years to provide feedback as an expert health care provider on developing the content and format of the fatigue self-management SMS text messaging intervention.

### Procedures

#### Overview

This study followed a user-centric co-design approach, meaning that participants helped to shape the intervention and their study experience [36]. We used step-by-step procedures to develop and test the utility of the fatigue self-management SMS text messaging intervention. The steps included the following: (1) development of the initial SMS text messaging library for the self-management intervention, (2) review and modification of SMS text messages, (3) integration of the content library into the SMS text messaging system, and (4) utility testing of the SMS text messaging prototype.

#### Step 1: Development of Initial Text Messaging Library for Fatigue Self-management

The investigator team formed an initial library of SMS text messages based on evidence-based resources from the National MS Society [37,38], the American Stroke Association [39], and the National Spinal Cord Injury Association [40]. The content collected was assessed by the research team and categorized into 9 key content areas for the library of targeted fatigue self-management SMS text messages (eg, sleep, energy conservation, and simplifying activities). The investigator team, composed of 3 licensed occupational therapists, also reviewed the content for accuracy and appropriateness. The investigator team further customized SMS text messages into patient activation measure (PAM) levels 1 to 2 and 3 to 4, where level 1 to 2 SMS text messages focused on building knowledge and increasing awareness and level 3 to 4 SMS text messages focused on increasing skills or maintaining behaviors for self-management [41]. The 4 PAM levels were collapsed into 2 levels to balance tailoring with feasibility (ie, the development of 2 series of SMS text messages was more achievable than that of 4, given our study time frame and funding). Furthermore, collapsing of the PAM levels has been suggested in previous studies based on psychometric assessments.

#### Step 2: Review and Modification of SMS Text Messages

##### Health Care Expert Opinion

A physiatrist with extensive care experience with persons with disabilities reviewed the SMS text messaging library for accuracy and appropriateness and participated in an in-depth interview conducted by the investigator team to determine the SMS text messaging library content customized to the fatigue self-management needs of individuals with MS, SCI, and stroke. The interview lasted approximately 1.5 hours and was conducted according to a discussion guide iteratively developed by the investigator team. Detailed notes were taken and summarized during the interviews. The investigator team refined the SMS text messaging library content based on the summary notes.

##### CAB Feedback

The investigator team identified, screened, and consented 6 participants to join the CAB. A total of 2 advisory board sessions were held via Zoom videoconferencing (Zoom Video Communications) for approximately 90 minutes each to solicit their feedback on the refined SMS text messaging library program. A session moderator, a note taker, and 2 additional research team members helped to facilitate these sessions. Before the first meeting, the investigator team sent a document displaying all the potential SMS text messages included in the program to the participants. Participants were asked to rate the clarity and relevance of all SMS text messages on a 5-point Likert scale (with 5 being the highest rating). The document also included a section in which participants could note their overall thoughts or explain their ratings of certain SMS text messages. During the meeting, the investigator team provided all the participants with background information about the project and patient activation. Subsequently, the investigator team elicited feedback regarding general thoughts and concerns about the refined SMS text messaging library program. The first half of the potential SMS text messages was discussed in the first session, and the remaining half of the SMS text messages was discussed in the second meeting. During the second meeting, the research team also asked the participants about the delivery of SMS text messages, such as the number of SMS text messages per day that would be appropriate and the best time to send them (morning or afternoon). Owing to scheduling conflicts, the first meeting was held twice (first time with 2 participants and repeated for the second time with 4 participants). Both sessions were audio-recorded and transcribed verbatim using Trint [42], a professional transcription service. The transcripts were checked for accuracy by members of the research team.

The original text library began with 57 SMS text messages. Following consultation with the physiatrist, we added the ninth content category of “fatigue education and awareness” and added 16 SMS text messages (for a total of 73 SMS text messages). Further refinement was made to the text library based on the CAB’s clarity and relevance ratings of each text on a 5-point Likert scale. If the mean relevance or clarity rating for a text was between 3.51 and 3.99, the investigator team modified the SMS text message to improve the phrasing or level of detail. If the mean relevance or clarity rating for a text was ≤3.5, the text was removed, leading to 61 SMS text messages. The investigator team finalized the text library with the highest-rated texts across each category. The final 48 texts also aligned with the CAB’s recommendation of 1 text per day during the 12 weeks (ie, Monday through Thursday only, 1 text per day for a total of 4 texts per week, with a question asking about the application of text content delivered on Friday). The number of texts was kept consistent across PAM levels 1 to 2 and 3 to 4.

#### Step 3: Integration of the Content Library Into the Text Messaging System

We used a digital platform designed by Epharmix (Epharmix, Inc) [43] to deliver the SMS text messaging intervention. We used the predeveloped intervention builder from Epharmix to set up the SMS text messaging logic. A research staff member programmed the SMS text messages and intervention logistics into the Epharmix system. Epharmix complementarily provided the intervention builder and some basic technical assistance. Information was sent and collected via SMS text messages, transmitted between the cellular carrier and the intervention builder using the Secure Sockets Layer, and stored in a secure and encrypted university MySQL [44] database server environment. A business associate’s agreement between Epharmix and the university was set up to ensure that electronic patient health information was kept secure. The investigators accessed the database using the Epharmix website.

**Figure 1:**
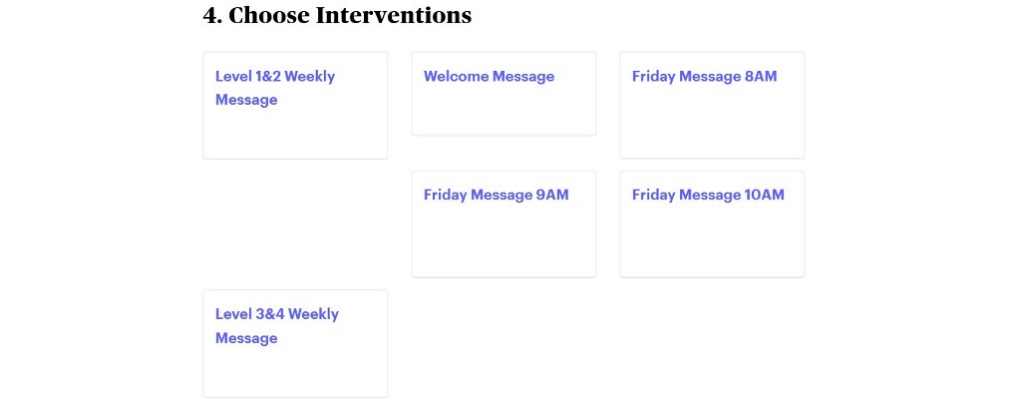
Available message prescriptions for participants.

**Figure 2:**
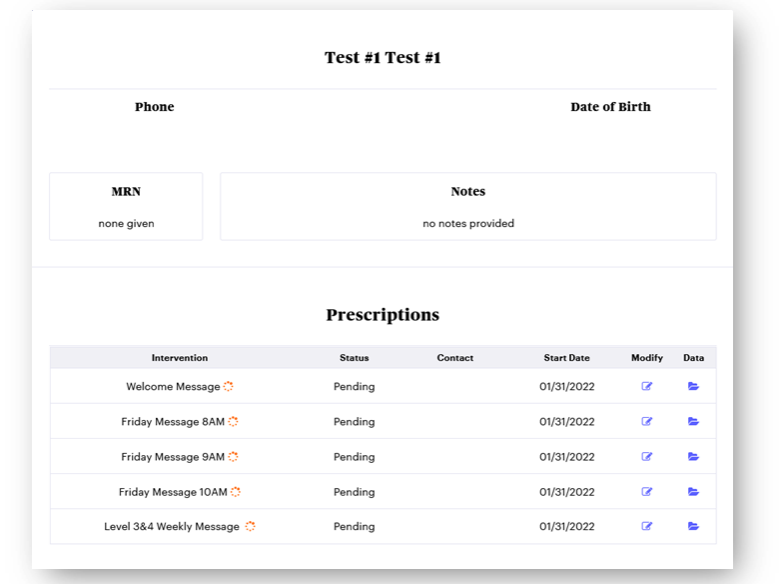
Participant dashboard. MRN: medical record number.

**Figure 3:**
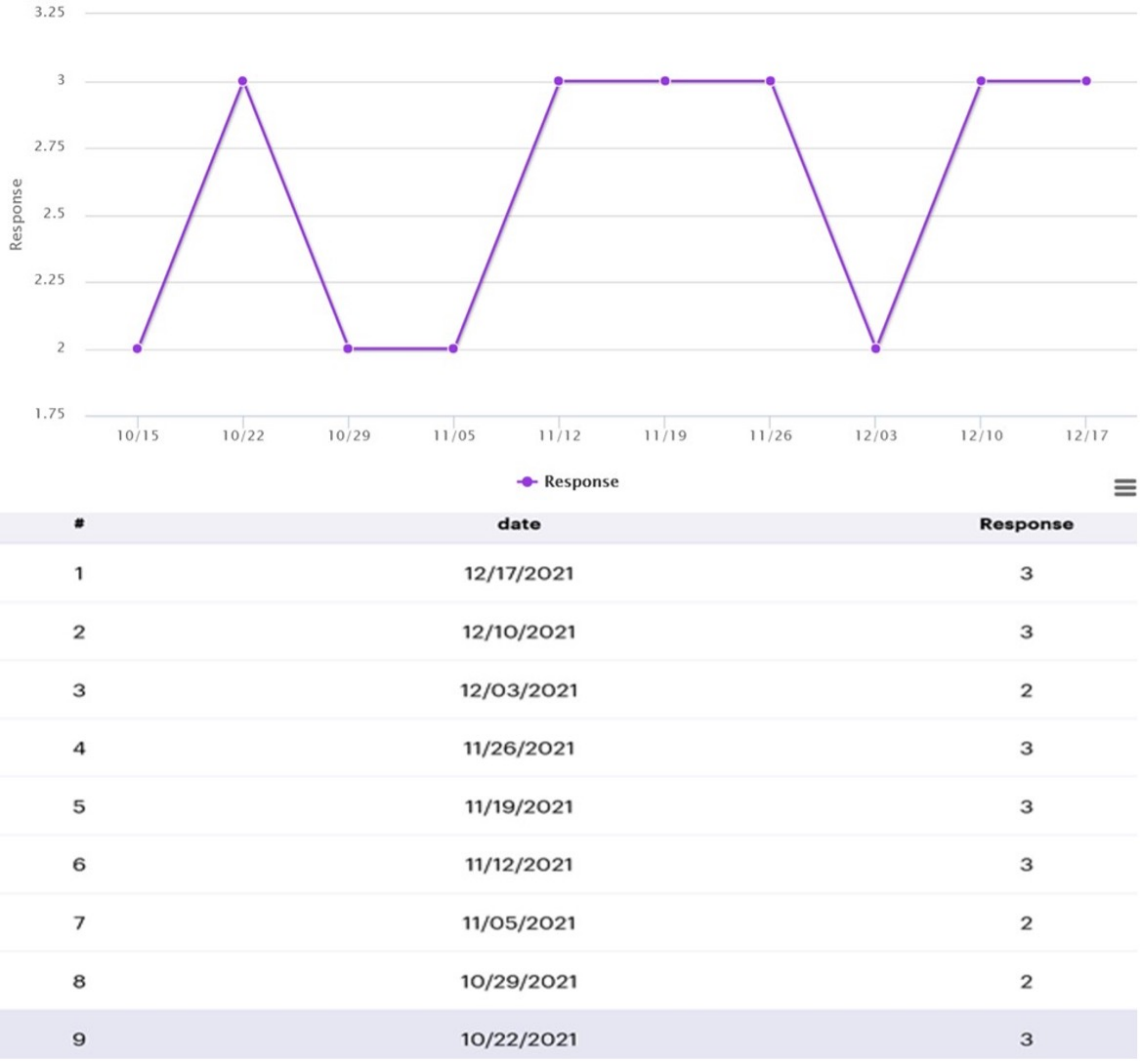
Participant responses to posed weekly question.

**Figure 4:**
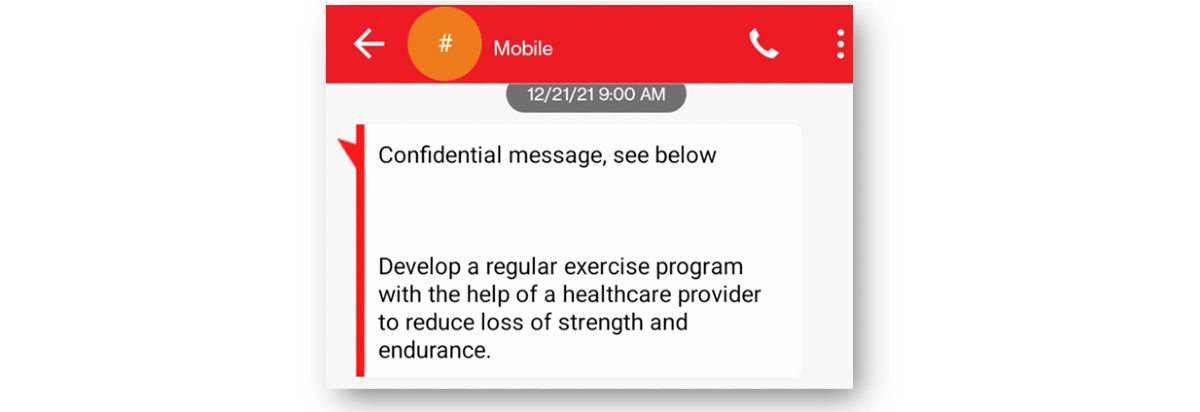
Example of daily fatigue management tip.

**Figure 5:**
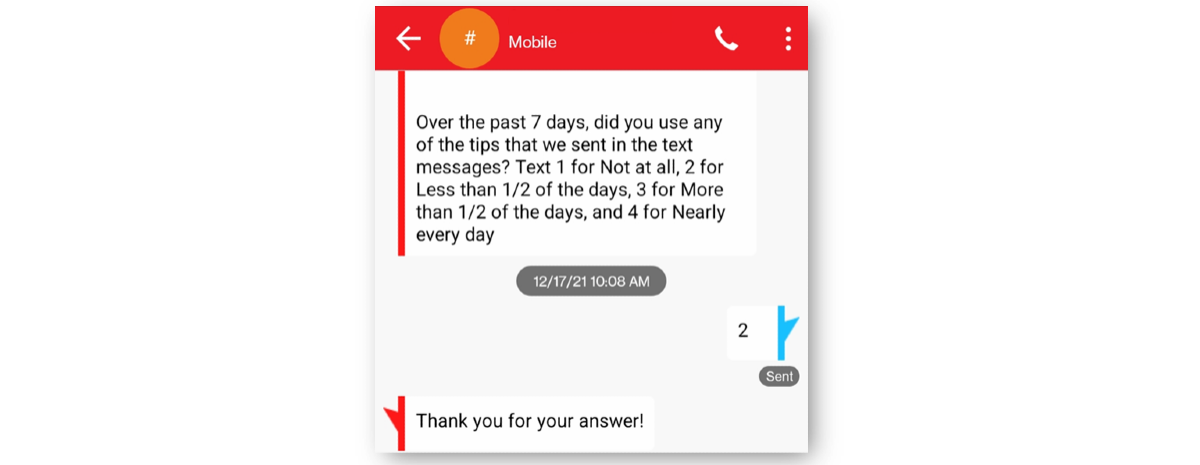
Example of posed weekly question and response.

In the Epharmix system, SMS text messages were grouped into message types, including a welcome message, PAM level 1 to 2 weekly SMS text messages, PAM level 3 to 4 weekly SMS text messages, and check-in SMS text messages asking participants to rate the impact of their fatigue on their daily life and report their use of the tips that week (Figure 1). The system allows research staff to examine participants’ information and monitor their responses to the check-in SMS text messages via the dashboard (Figures 2 and 3). Figures 4 and 5 show screenshots of how the SMS text messages were displayed on a mobile device.

#### Step 4: Utility Testing of the Text Messaging Prototype

Once the entire fatigue management SMS text messaging intervention was programmed into the Epharmix intervention builder platform, the 6 CAB members tested the SMS text messaging prototype by receiving SMS text messages on their mobile devices for 1 week. The investigator team sent a usability survey to all CAB members immediately after they completed the 1-week trial. The survey consisted of 11 questions with a 1-7 Likert scale response (1=strongly disagree to 7=strongly agree) and 5 additional qualitative questions. Qualitative questions included whether CAB members thought the timing of the SMS text messages was appropriate, whether they felt that the frequency of the SMS text messages sent was appropriate, whether they had any difficulties responding to the check-in questions, whether they had any further comments regarding the SMS text messaging intervention, and whether they approved of the SMS text messaging intervention. Quantitative questions on the usability survey were adapted from the extended version of the Technology Acceptance Model, which has been shown to accurately account for a large percentage of variance in user usefulness perceptions [45].

### Data Analysis

In step 2, the research team reviewed detailed notes from the expert physician, identified recommendations that aligned with the goals of the intervention, and made the version for the CAB to review. The CAB member ratings for each SMS text message were also collected and combined into 1 master spreadsheet for analysis. The investigator team computed the mean clarity and relevance ratings and identified the highest-rated and lowest-rated SMS text messages. Transcriptions of the advisory board sessions produced by Trint were checked for accuracy by 2 trained graduate students. Using a content analysis approach [46], the investigator team deductively developed a formal codebook based on responses from the semistructured questions. A total of 2 graduate students used the codebook to independently code all transcribed texts and met weekly to discuss and reconcile coding discrepancies. In addition, the advisory board qualitative data were triangulated with the data from the usability survey and the physician’s input, and establishing interrater reliability was therefore deemed as unnecessary [47]. We summarized the feedback into the final coding results, which were incorporated into the modifications made to the final version of the text messaging prototype. We used NVivo (version 12; QSR International), a qualitative data analysis software, to analyze the qualitative data [48]. In step 4, all CAB utility ratings were tracked in a spreadsheet and reviewed for the frequency of ratings. Comments shared in the open-ended questions were reviewed for themes.

### Ethics Approval

Institutional review board approval from Washington University in St. Louis was obtained before the study (20210319).

### Consent, Data Security, and Compensation

All participants were provided with a letter of information, considered a waiver of written consent, before participating, which described the details and purpose of the study. Study data were deidentified and collected through secure and Health Insurance Portability and Accountability Act–compliant platforms (eg, university email and videoconferencing). Participants were financially compensated US $50 per CAB meeting for up to 4 meetings in the form of a written check that was mailed to them.

## Results

### Step 1: Development of Initial Text Messaging Library for Fatigue Self-management

A total of 9 focus areas (8 identified by the research team and 1 added after consultation with the physiatrist) were developed to be delivered over 12 weeks. A total of 57 SMS text messages were initially created for PAM levels 1 to 2 and 3 to 4 (Tables 1 and 2) from the current evidence and resources on fatigue management. The 57 SMS text messages were increased to 73 SMS text messages following consultation with the physiatrist, refined to 61 after the CAB review, and decreased to 48 by the investigator team to fit within the 12-week time frame (ie, 1 text per weekday, Monday through Thursday only, for a total of 4 texts per week).

**Table 1:** Summary of SMS text category development and number of SMS text messages.

Weekly topic	Operational definition	Number of text messages			
		Original content development	After a physiatrist consult	After consumer advisory board reviews	Final
Fatigue education and awareness (week 1)^a^	Learn about fatigue and how to be individually aware of its impact, as it is different for each person	0	6	5	4
Behavioral techniques (weeks 2 and 3)	Change certain behaviors to reduce fatigue	14	16	11	8
Energy conservation (weeks 4 and 5)	Prevent wasteful use of energy to minimize fatigue in daily activities	7	8	8	8
Environment and assistive technology (week 6)	Alter your surroundings and use adaptive equipment to preserve energy and decrease fatigue	8	8	6	4
Simplifying activities (week 7)	Break down chores and tasks into smaller pieces so that they are easier to manage and do not cause greater fatigue	6	6	4	4
Diet and hydration (week 8)	Understand the impact that hydration and healthy food can have on fatigue	3	6	6	4
Effective communication (week 9)	Ensure that the people around you listen and understand your ideas and concepts regarding how fatigue impacts you	4	5	4	4
Exercise and physical activity (weeks 10 and 11)	Determine the right amount and type of exercise or physical activity to combat fatigue	10	11	10	4
Sleep (week 12)	Develop healthy sleep patterns to decrease fatigue	5	7	7	8
Total SMS text messages for all patient activation measure levels 1-4	N/A^b^	57	73	61	48^c^

^a^This topic was added following consultation with the physiatrist; before that, we only had 8 topics. The number of SMS text messages was kept consistent across the patient activation measure levels 1 to 2 and 3 to 4.

^b^N/A: not applicable.

^c^One text per day, Monday through Thursday only, a total of 4 SMS text messages per week for 12 weeks.

**Table 2:** Examples of SMS text messages by patient activation measure levels.

Weekly topic	Example SMS text messages for PAM^a^ levels 1-2	Example SMS text messages for PAM levels 3-4
Fatigue education and awareness (week 1)	Recognize that there can be different causes of fatigue, such as pain, daily activities, and stress. Figuring out what causes your fatigue can be a helpful step in learning how to manage it.	Maintain communication with your health professional to learn how to recognize what is causing your fatigue.
Behavioral techniques (weeks 2 and 3)	Recognize that there are different types of fatigue (such as physical, emotional, and mental or cognitive). Understanding different types of fatigue can help you manage it.	Talk to a health professional about your fatigue. They can help you find strategies to manage symptoms associated with your type of fatigue.
Energy conservation (weeks 4 and 5)	Ask for help with tiring activities. Tell family or friends if you need help with tasks that are difficult for you.	Keep track of which activities fatigue you and plan those activities during the day when you typically have more energy. This will allow you to complete more tasks throughout the day.
Environment and assistive technology (week 6)	Learn about different assistive technology options and ergonomic techniques that can help conserve energy.	Consider using one or more assistive technology or ergonomic technique that works best for you. This can help to reduce strain and stress on your body.
Simplifying activities (week 7)	Changing the way you do certain activities or changing your expectations of yourself can give you more energy.	Think about ways your fatigue can be managed. Try simplifying daily tasks in keeping with your capabilities to manage fatigue.
Diet and hydration (week 8)	Recognize that nutrition plays a role in combating fatigue. Learn about healthy diet choices that can impact energy levels.	Eating a diet with whole grains, nuts, seeds, and lean proteins will keep your body fueled regularly and help you beat fatigue.
Effective communication (week 9)	Talk with family and friends about your fatigue and be honest about how it impacts your daily life. This reality may also make you more aware of your own fatigue.	Make an effort to honestly communicate with your family and friends about how fatigue impacts your daily life consistently (maybe schedule a weekly check-in).
Exercise and physical activity (weeks 10 and 11)	Realize that a regular exercise routine can reduce loss of strength and endurance and improve fatigue symptoms.	Consistently set an exercise goal and plan it into your weekly schedule. This should make following it easier.
Sleep (week 12)	Treat symptoms that may interfere with sleep, such as spasticity or urinary problems. This can help to reduce fatigue.	Speak with your health care provider about any symptoms, such as spasticity or urinary problems, that may be interfering with your sleep and increasing your fatigue.

^a^PAM: patient activation measure.

### Step 2: Review and Modification of SMS Text Messages

#### Health Care Expert Opinion

Modifications based on the expert physician’s opinion were made to address two main points: (1) the SMS text messaging intervention should introduce basic fatigue education in its first week and (2) the wording of SMS text messages and the sources from which the content is developed should be more “mainstream.” The first point led to the addition of “fatigue education and awareness” as a new category placed at the beginning of the program to educate about fatigue, different types of fatigue, and their causes, resulting in 9 focus areas. The second point led to the review of more mainstream sources for examples of a layman’s presentation and wording of evidence-based information on fatigue (eg, *Time* magazine and *Men’s Health* magazine) and an improvement in the readability of the SMS text messages.

#### CAB Feedback

A total of 6 persons with disabilities participated as advisory board members to provide feedback on the SMS text messaging intervention. The advisory board was evenly divided by sex and reported an average age of 47 (SD 13) years (Table 3). Regarding the quantitative feedback, the range in relevance ratings for individual SMS text messages was from 3.2 to 5.0, and the range in clarity ratings was from 3.5 to 5.0 across 9 focus areas. Table 4 outlines a range of quantitative ratings for various example SMS text messages, accompanied by the action taken (ie, remove, modify, or keep).

We summarized the qualitative advisory board feedback based on the 9 SMS text message focus areas (ie, fatigue education and awareness, behavioral techniques, energy conservation, environment and assistive technology, simplifying activities, diet and hydration, effective communication, exercise and physical activity, and sleep; Table 5). Participants provided a wide range of qualitative feedback related to the clarity and relevance of the SMS text messages. The participants also described their personal experiences with some tips or suggestions in the SMS text messages they used for fatigue self-management. A common theme expressed by participants across all areas was the need for more examples (eg, for the diet and hydration area, participants explained that examples of healthy snacks would be helpful). Overall, participants reported that the SMS text messaging content was helpful, clear, and well suited for a mHealth intervention for persons with MS, SCI, and stroke. The advisory board noted that the content seemed particularly relevant for newly diagnosed individuals, as some of the information was not new knowledge for them and served as more of a reminder.

**Table 3:** Overview of advisory board demographics (N=6).

Participant characteristics		Value
Age (years), mean (SD; range)		46.67 (12.6; 25-59)
**Gender, n (%)**		
	Male	3 (50)
	Female	3 (50)
**Race, n (%)**		
	Black or African American	1 (17)
	White	5 (83)
**Ethnicity, n (%)**		
	Hispanic or Latino origin	0 (0)
	Non-Hispanic or Latino origin	6 (100)
**Diagnosis, n (%)**		
	Multiple sclerosis	2 (33)
	Spinal cord injury	2 (33)
	Stroke	2 (33)

**Table 4:** Examples of the range of quantitative advisory board feedback and changes made.

Content categories	Initial SMS text message example	Clarity rating (1-5), mean (SD)	Relevance rating (1-5), mean (SD)	Action taken
Fatigue education and awareness	“Take your fatigue medication on time because sometimes fatigue can occur without any warning.” (PAM^a^ level 1-2)	5.0 (0.7)	3.2 (0.1)	Removed
Behavioral techniques	“Participate in cognitive behavioral therapy (CBT) to cope with difficult situations with the help of a health care provider.” (PAM level 3-4)	4.2 (0.5)	3.8 (0.3)	Modified by replacing “cognitive behavioral therapy” with “problem-solving skills”
Energy conservation	“Use grocery delivery services to conserve energy that would be spent going to the store.” (PAM level 1-2)	4.7 (0.3)	3.8 (0.4)	Modified so that grocery delivery is an example rather than a focus of the text
Environment and assistive technology	“Plan ahead for your return back to work or school and think about modifying activities and tasks that are more difficult so that you can continue to do them.” (PAM level 3-4)	3.5 (0.6)	3.8 (0.6)	Removed
Simplifying activities	“Taking rest breaks between demanding tasks can help you combat fatigue.” (PAM level 1-2)	4.8 (0.4)	4.7 (0.5)	Kept
Diet and hydration	“Eat healthy foods that are high in iron. Plan to add these food choices to your meal to promote more long-term energy: spinach, legumes, pumpkin seeds, turkey, broccoli, tofu, & fish.” (PAM level 3-4)	5.0 (0.1)	4.7 (0.4)	Kept
Effective communication	“Recognize that joining a support group can help to decrease your frustration with having long-term fatigue.” (PAM level 1-2)	4.3 (0.5)	4.2 (0.4)	Kept
Exercise and physical activity	“Active wheeling or walking to places you would have normally traveled to in a vehicle or by parking a bit farther away will provide some exercise or physical activity.” (PAM level 1-2)	4.2 (0.3)	3.8 (0.4)	Modified by replacing reference to parking with a general statement about using active wheeling or walking instead of driving
Sleep	“Monitor the temperature of your sleeping space to make sure it is not too hot. Heat can make it more difficult to fall and stay asleep and can increase fatigue.” (PAM level 3-4)	4.7 (0.1)	5 (0.5)	Kept

^a^PAM: patient activation measure.

**Table 5:** Examples of qualitative advisory board feedback.

Content category	Initial SMS text example	Summarized feedback	Example quotes
Fatigue education and awareness	“Figure out which type of fatigue you may be experiencing by talking to a health professional. Your fatigue may look different from other people’s fatigue, and they can help you figure out what works best for you.” [PAM^a^ level 3-4]	This statement should be delivered earlier in the week.	“This statement should come earlier.” [58-year-old White woman]
Behavioral techniques	“Schedule meaningful activities into your daily routine to help fight fatigue.” [PAM level 3-4]	The phrase “meaningful activities” is confusing and vague.	“What are some examples...that would be meaningful to somebody that would help them reduce fatigue? Because I had a hard time. If I sat down on the sofa and watched TV all afternoon, I might reduce my fatigue, but I don’t think that’s good for anybody.” [44-year-old White woman]
Energy conservation	“Planning visits and knowing what challenges you will face when arriving will help to manage energy use.” [PAM level 1-2]	The term “visits” is too vague.	“Can you be a bit more specific than ‘visits’?” [58-year-old White woman]
Environment and assistive technology	“Be aware that different body positions can cause strain, which may increase feelings of fatigue.” [PAM level 1-2]	Something we just figure out on our own.	“It just didn’t seem like it really was relevant because it’s like, you kind of live it and you’ve kind of just got to test out the waters to see what works good for you.” [37-year-old Black or African American man]
Simplifying activities	“Reducing the number of transfers you make daily can help you to reduce your fatigue.” [PAM level 1-2]	The text needs to specify to whom transferring is relevant.	“Can you specify what kind of person needs to be concerned about transferring?” [58-year-old White woman]
Diet and hydration	“Eat a balanced diet regularly to reduce fatigue.” [PAM level 1-2]	Examples would be helpful.	“Perhaps include a list of healthy snack ideas. Sometimes people with disabilities are too fatigued to eat full meals.” [57-year-old White woman]
Effective communication	“Talk with family and friends about your fatigue and be honest about how it impacts your daily life. This reality may also make you more aware of your own fatigue.” [PAM level 1-2]	This text message tip is easier said than done.	“This would be a delicate subject, talking to family and friends about fatigue. Other people usually think sitting down for 10 minutes is all I need. They have no frame of reference.” [57-year-old White woman]
Exercise and physical activity	“Find self-care activities that are important to you. This will help to reduce your fatigue levels.” [PAM level 1-2]	Examples would be helpful.	“Pretty vague. Do you mean putting on makeup and styling your hair?” [57-year-old White woman]
Sleep	“Electronic devices’ artificial blue light can suppress the sleep hormone melatonin, which may make it more difficult for you to fall asleep. Track your screen time throughout the day to determine if it makes a difference in your sleep.” [PAM level 1-2]	The 2 statements need a clearer connection between each other.	“I don’t see the correlation between the first and second sentence. It’s the second one that impacts sleep.” [58-year-old White woman]

^a^PAM: patient activation measure.

Table 6 lists the additional delivery and logistics codes with example quotes. The participants agreed that SMS text messages should be grouped by topic for each week. They also agreed that SMS text messages should be delivered at the same time every day, preferably in the morning. Regarding personalization of the SMS text message content, participants felt that SMS text messages with introductory content, reminders, and general statements would be best suited for individuals with a newly diagnosed disability. In contrast, the content should be more personalized, tailored, and strategy based for those who have lived with their disability for a longer period. The CAB has also reached a consensus on the preference for only 1 SMS text message per day. This feedback led the investigator team to narrow down the program to deliver 1 SMS text message per day for 12 weeks. Therefore, the research team eliminated the SMS text messages with the lowest relevance and clarity ratings.

**Table 6:** Examples of delivery and logistics feedback.

Delivery and logistics codes	Topic description	Consensus	Example quote
Format	In what order the content should be delivered	Structured and grouped together	“I like it more structured, too, where, you know, you can be like, every Wednesday, you get something about working out or stuff like that or, ‘did you work out today?’” [57-year-old White woman]
Personalization	How personalized the content should be	Broad and simple is better for newly diagnosed individuals; more personalized and strategy based is better for individuals who have lived with their disability for a longer period	“If you just had a stroke or you were just diagnosed with MS or you just got a spinal cord injury, then you know, this is all great. But if you’re further along, like, I don’t need a reminder to take medicine, and I don’t want it either.” [58-year-old White woman]
Timing	At what time the content should be delivered	Once per day in the morning	“I think if they were at the same time every day, it would be more coordinated. Sometime in the morning.” [59-year-old White man]

### Step 3: Integration of the Content Library Into the Text Messaging System

In most cases, research and Epharmix staff worked together and obtained the functionality that the designed SMS text messaging intervention required. However, we encountered a few limitations with programming. The first limitation incurred during programming was that the intervention builder application only allowed 50 scripts (the individual SMS text messages sent to the users), but the designed SMS text messaging intervention required 135 scripts. The Epharmix staff were able to open the permissions and allow up to 200 scripts.

The next limitation was that the Epharmix allowed only 3 different sets of SMS text messages. However, this project had 6 different sets of scripts to deliver the 12-week fatigue self-management SMS text messaging intervention. The first set was a single message delivered once as a welcome message to all the participants before beginning the intervention phase. The second set of scripts consisted of 48 separate daily fatigue management tips delivered at the same preset time once a day on Monday through Thursday for participants who scored a PAM level of 1 to 2. The third set of scripts was a different set of 48 daily fatigue management tips delivered at a preset time once a day on Monday through Thursday for participants who scored a PAM-13 level of 3 to 4. Scripts fourth to sixth were single SMS text messages set up to deliver check-ins on Fridays at 3 different time points and with 3 different SMS text messages. The first Friday message posed a question asking the participants to rate how their fatigue had impacted their daily life over the past week (1=not at all to 5=very much). The second message asked the participants to rate how often they had used the provided fatigue tips over the past week (1=not at all to 4=nearly every day). The third message was a motivational one that thanked them for their participation and commended them on a job well done. The Epharmix staff was able to open permissions, allowing the system to accept 6 sets of SMS text messages.

The next set of challenges came during the research staff and CAB testing of the developed SMS text messaging intervention. Every SMS text message delivered began with a “confidential message, see below.” The wording confused CAB members, and they did not know who was sending the SMS text messages. Unfortunately, the SMS text message was automatically generated by the system, and it could not be deleted or edited. We also discovered that the SMS text messages sent to participants did not come from the same phone number, which made the program’s SMS text messages harder to identify as being related to the study. Therefore, we decided to educate all research participants before the intervention phase that SMS text messages would come from different phone numbers, and they would always begin with the “confidential message” wording.

Several more limitations of the intervention builder application did not necessarily impact the participants but influenced how we managed the study. We could not repeat the SMS text messages if the participant did not respond, and there was no way for us to know whether the SMS text messages were opened or read. To overcome these limitations, the research coordinator planned to closely follow the required weekly responses and conduct follow-up phone calls during the next testing phase with future research participants in the full 12-week program.

### Step 4: Utility Testing of the Text Messaging Prototype

After completing the 1-week trial of the SMS text messaging intervention, the CAB members provided feedback on their experiences using the usability survey. Members rated the first 11 questions of the survey using a 1-7 Likert rating scale. Table 7 presents the questions and responses. The CAB members supported the developed formatting and usability of SMS text messaging. There was a single rating of 1, which was related to the question, “Whenever I made a mistake using the text messaging program, I could recover easily and quickly.” The CAB member who reported this rating commented, “I’m sure it’s easy to recover from a mistake—hit the back key on my phone and was able to answer the question—but I’m not sure if this was the correct thing to do. I most likely missed the instruction—what to do if you make a mistake.” The low rating was not necessarily because the participant disagreed with the ease of recovering from a mistake but because they were unsure whether the method they used was correct.

**Table 7:** Advisory board member perspectives on usability (scale of 1=strongly disagree to 7=strongly agree, or not applicable).

Usability statement	Value, mean (SD)
The SMS (text) program format was easy to use	7 (0)
It was easy for me to learn to use the SMS (text) program	7 (0)
The questions posed in the SMS (text) program were easy to read, understand, and respond to	6.5 (0.8)
Whenever I made a mistake using the SMS (text) program, I could recover easily and quickly (missing=3)	5 (3.5)
I like the SMS (text) program	7 (0)
The information in the SMS (text) program was well organized	6.7 (0.8)
I feel comfortable using the SMS (text) program in social settings	7 (0)
The amount of time involved in using the SMS (text) program has been fitting for me	7 (0)
I would use the SMS (text) program again	7 (0)
Overall, I am satisfied with the SMS (text) program	7 (0)
The SMS (text) program has all the functions and capabilities I expected it to have	6.8 (0.4)

The 5 additional open-ended questions in the usability survey primarily received positive feedback, supporting the usability of the SMS text messaging intervention. The first question, “Did you feel like the timing (in the morning) of the daily text messages was appropriate?” received responses including the following: “fit my schedule well,” “I work nights, but I found the messages when I woke up,” and “it was the middle of my workday or seemed like it—but I work 6 am to 3 pm, more than likely it’s OK for everyone.” The second question, “Did you feel like the frequency of how often the text messages were sent was appropriate” received a single response: “A couple more would have been appropriate also, like maybe in the evening.” The next question, “Did you have any difficulties responding to the check-in questions posed on Fridays,” also received a single response: “The number 1 was already entered as the response, and the first time, I accidentally sent that as my response. The second time, I was able to send my own response.” The fourth question, “Do you approve of the text program format that has been developed” received 2 responses: “Did a nice job, liked all the content*,*” and “I liked how y’all developed it—informative but not overwhelming.”

## Discussion

### Principal Findings

Fatigue is a common chronic condition for persons with disabilities [49]. Education on fatigue self-management is crucial for supporting patient activation to reduce fatigue [50]. This study described the 4-step process used to develop the fatigue self-management SMS text messaging intervention. The expert physician and CAB provided insight into the clarity, relevance, and content of the fatigue self-management SMS text messaging intervention for persons with MS, SCI, and stroke. Participants in the CAB found the content of the SMS text messages relevant, providing support, motivation, and accountability via a simple and convenient mode of communication. Participants also indicated that 1 weekly check-in message and 4 fatigue self-management SMS text messages per week were appropriate for intervention delivery. Our findings concur with those of an earlier qualitative study; participants in both studies showed positive experiences with SMS text messaging interventions and perceived the incorporation of SMS text messages as an adjunctive tool to support health management [51]. Previous mHealth SMS text message–based interventions have used a variety of methods to develop message content. Some recent mHealth interventions have used message libraries adapted from previous successful trials or developed based on feedback from expert working groups without consultation from end users [52,53]. However, recent studies have incorporated co-design or participatory methods, and this approach appears to be becoming the standard. Examples of end user or consumer involvement in the development stages include real-time ratings, daily qualitative telephone interviews, crowdsourcing, usability testing, and mixed co-design workshops comprising both health professionals and consumers [27,54-56].

This study incorporated the concept of patient activation into the development of the fatigue self-management SMS text messaging intervention for individuals with various disabilities. This approach is similar to another ongoing trial in Southeast Asia that proposed the development and testing of a mobile app–based self-management program to empower people with knowledge and skills to manage metabolic syndromes (eg, diabetes or hypertension) [57]. A unique finding of our study is that our process involved both medical staff and persons with disabilities as part of the team to guide the development of the digital intervention. Participants also evaluated all SMS text messages to increase their relevance and clarity before the SMS text messages were incorporated into the digital platform. Participants made further suggestions to support intervention delivery and improve user engagement. One suggestion was to provide a structured format wherein we would send SMS text messages at the same time throughout the intervention. We incorporated this suggestion by programming all SMS text messages to be presented in a structured sequence. Another suggestion was to personalize SMS text messages based on the chronicity of an individual’s disability. Interventions for persons with a recent onset of disease or following a new injury should emphasize psychoeducation, which enables people to learn and adjust to a new disability. In contrast, interventions for people in the chronic stage should emphasize building strategies to cope with barriers encountered while living in the community [58,59]. Our SMS text messaging intervention is focused on increasing self-management knowledge and skills by providing patient activation–based health tips to manage fatigue. This intervention would likely be appropriate for persons with various chronic stages of disability. Further research is required to support this argument.

Challenges were encountered when integrating SMS text messages into the digital platform. A key lesson learned in this study is to foster close collaboration between the technology industry and medicine to develop or adapt technology [60]. We also confirmed the importance of involving the end user to help guide the development and optimization of the technology to be used in medicine. This practice should become standard both within health care and across many other sectors. With support from our technology partner, we could adapt several features of intervention technology to meet the needs of our target population without sacrificing functionality. We also conducted usability testing in which our CAB members trialed the SMS text messaging intervention for a week. They endorsed all items with positive responses, suggesting the adequate usability of the SMS text messaging intervention. Future research is needed to examine the usability of this program in a larger disability group.

### Additional Study Limitations

A potential limitation is that our SMS text messaging intervention included SMS text messages from 9 domain areas. It is unclear which domain areas are most effective. A future trial may ask participants to rate which domains of SMS text messages they find most helpful in managing their fatigue. We designed the intervention content by structuring various domain areas across 12 weeks. We anticipate that not all content will benefit all participants at one point in time. A future study may explore whether adding a preparation session to identify the areas needed and prescribing the relevant SMS text messages for each participant would lead to a better outcome.

### Conclusions

This study demonstrates a robust method for developing a fatigue self-management SMS text messaging intervention for people with MS, SCI, and stroke. With input and support from a medical expert, persons with disabilities, and the technology vendor, we developed an intervention delivered through technology to people with various disabilities at different patient activation levels. The next steps include pilot-testing the fatigue self-management SMS text messaging intervention with people with MS, SCI, and stroke to examine its flexibility and explore its initial effects.

## Abbreviations

CAB: consumer advisory board
mHealth: mobile health
MS: multiple sclerosis
PAM: patient activation measure
SCI: spinal cord injury

## Data Availability

The underlying data sets generated and analyzed during this study cannot be sufficiently deidentified and, therefore, cannot be made publicly available because of ethical considerations. Deidentified data can be made available from the corresponding author upon reasonable request for the purpose of further research.
